# Cadherin Cell Adhesion System in Canine Mammary Cancer: A Review

**DOI:** 10.1155/2012/357187

**Published:** 2012-08-22

**Authors:** Adelina Gama, Fernando Schmitt

**Affiliations:** ^1^Centro de Ciência Animal e Veterinária (CECAV), Universidade de Trás-os-Montes e Alto Douro (UTAD), 5001-911 Vila Real, Portugal; ^2^Institute of Molecular Pathology and Immunology of the University of Porto (IPATIMUP), 4200-465 Porto, Portugal; ^3^Medical Faculty, University of Porto, 4200 Porto, Portugal

## Abstract

Cadherin-catenin adhesion complexes play important roles by providing cell-cell adhesion and communication in different organ systems. Abnormal expression of cadherin adhesion molecules constitutes a common phenomenon in canine mammary cancer and has been frequently implicated in tumour progression. This paper summarizes the current knowledge on cadherin/catenin adhesion molecules (E-cadherin, **β**-catenin, and P-cadherin) in canine mammary cancer, focusing on the putative biological functions and clinical significance of these molecules in this disease. This paper highlights the need for further research studies in this setting in order to elucidate the role of these adhesion molecules during tumour progression and metastasis.

## 1. Introduction

In canine species, spontaneous mammary tumours constitute the second most frequent neoplasia, surpassed only by skin tumours. When considering female dogs, mammary tumours represent the most common neoplasia, with malignant tumours accounting for up to 50% of cases [1]. Therefore, this disease represents a serious problem in worldwide veterinary practice and is a matter of concern for both oncologists and pathologists, which is ultimately reflected on the escalating number of studies in this research area. Furthermore, canine mammary tumours have attracted considerable attention over the years as possible animal models for human mammary neoplasia, based on their morphological and biological similarities [2–4].

Mammary tumours of the female dog are commonly associated with the development of distant metastases, which ultimately leads to morbidity and mortality. In the initial steps of this complex biological process, neoplastic cells lose intercellular adhesion in order to invade local tissues, and it is now evident that tumour invasion and progression may result from changes in cell adhesion systems. Based on sequence homology and structure, cell adhesion molecules are divided into the following families: cadherins, selectins, integrins, the immunoglobulin superfamily, and lymphocyte homing receptors, such as CD44 [5]. In this paper, we will discuss the findings on cadherin-mediated cell adhesion systems in canine mammary cancer, focusing on the putative biological functions and clinical significance of these molecules in this disease.

## 2. Overview of Cadherins

Cadherins are calcium-dependent cell-cell adhesion molecules, believed to be essential in coordinating morphogenetic cell movements and in the maintenance of normal tissue architecture [6–8]. Over the last two decades, cadherin research has been focused on its possible implication in general carcinogenic pathways, including human breast and canine mammary cancer, as well as in its putative involvement in tumour cell invasion and progression.

Until now, more than 20 members of the cadherin family have been described, characterized by cell type-specific expression patterns [8], with classical cadherins being the best characterised and most widely distributed members of the family [9].

**Figure 1:**
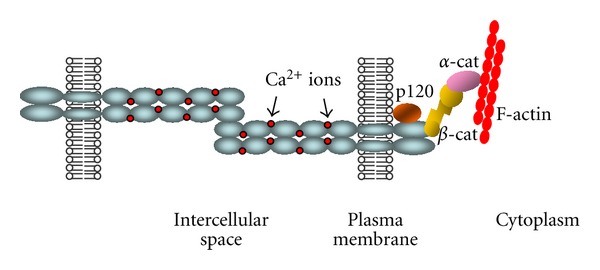
Schematic overview of the classical cadherin-catenin complex. Classical cadherins (blue), which mediate calcium-dependent (red) intercellular adhesion, are composed by an extracellular domain, a transmembrane domain and a cytoplasmic domain. This one comprises a juxtamembrane domain, which binds to p120-catenin (orange), and a catenin-binding domain, which binds *β*-catenin (yellow). *β*-catenin binds to *α*-catenin (violet), which establishes a direct link between the cadherin-catenin complex and the actin cytoskeleton (red).

Classical cadherins and their associated catenins form adhesion structures identified as adherens junctions [10]. Cadherins are single-pass transmembrane proteins comprising an extracellular domain, a transmembrane domain, and a cytoplasmic tail. The extracellular domain is composed of five cadherin repeats that are involved in promoting calcium-dependent homotypic cell-cell adhesion forming a zipper-like structure between neighbouring cells, while the intracellular domain interacts with cytoplasmic proteins termed catenins (*α*-, *β*-, *γ*-catenins), which link cadherins to the actin cytoskeleton (Figure 1) [10, 11].

Epithelial (E-) cadherin (also called uvomorulin, L-Cam, cell-Cam 120/80, or Arc-1) was the first to be identified and constitutes the prototypic member of the classical cadherin family. E-cadherin is a 120 kDa glycoprotein and is found in almost all epithelial tissues [12]. Other members include P-cadherin (placental cadherin) and N-cadherin (neuronal cadherin) [9, 13–15]. P-cadherin was originally found to be highly expressed in mouse placenta throughout pregnancy and is restricted to the basal or lower layers of adult stratified epithelium [9, 12], whereas N-cadherin is expressed by neuronal and muscle cells in human embryo and adult tissues [13].

The function and strength of cadherin-mediated adhesion depends on its dynamic association with catenins [16]. The cadherin cytoplasmic tail (catenin-binding domain, CBD) binds directly to *β*- and *γ*-catenin, which in turn binds to *α*-catenin to link the cadherin to the cytoskeleton, stabilizing the junctional structures [11, 17, 18].

Besides being a cytoplasmic protein involved in linking cadherin family receptors to the actin cytoskeleton [17, 19, 20], *β*-catenin is also a cotranscriptional activator of genes in the nucleus together with the lymphoid enhancer factor (LEF)/T-cell factor (TCF) [21–26], as a central component of the canonical Wnt signalling pathway [27]. Therefore, when free and stabilized in the cytoplasm, *β*-catenin promotes the activation of several genes, such as c-Myc and cyclin-D1, leading to changes in cell morphology, proliferation, and motility [28]. 

P120-catenin interacts directly with the juxtamembrane domain (JMD) of cytoplasmic tail and is regulated by tyrosine kinases, modulating cadherin intracellular trafficking, stability, adhesive capacity, and cell motility. Besides the interaction with p120 catenin, JMD supports lateral clustering and adhesive strengthening of cadherin complexes [29]. P120-catenin might modulate cell adhesion by influencing the organization of the actin cytoskeleton or by influencing the activity of RhoA, a small GTPase involved in actin cytoskeletal remodelling [30, 31].

Cell adhesion is a dynamic process regulated at various levels, including gene transcription, protein stability, and posttranslational modifications, in particular by phosphorylation of *β*-catenin and p120-catenin. Although intracellular signalling pathways (Wnt, TGF-*β*, MAPK) might affect cadherin-mediated adhesion, the establishment of adherens junctions also triggers signalling, which might involve Rho GTPases or growth factor receptors, such as EGFR [10]. The dynamic characteristics of the cadherin-catenin complex are important for embryogenesis and tissue repair, but can as well contribute to tumour development and progression [32], which has been scrutinized in detail, considering the amount of publications regarding this subject. In the following sections, the findings on cadherin-mediated cell adhesion systems in canine mammary cancer will be considered, as well as possible future directions in this spontaneous animal model.

## 3. Cadherin and Catenins in Normal Canine**** Mammary Gland

**Figure 2:**
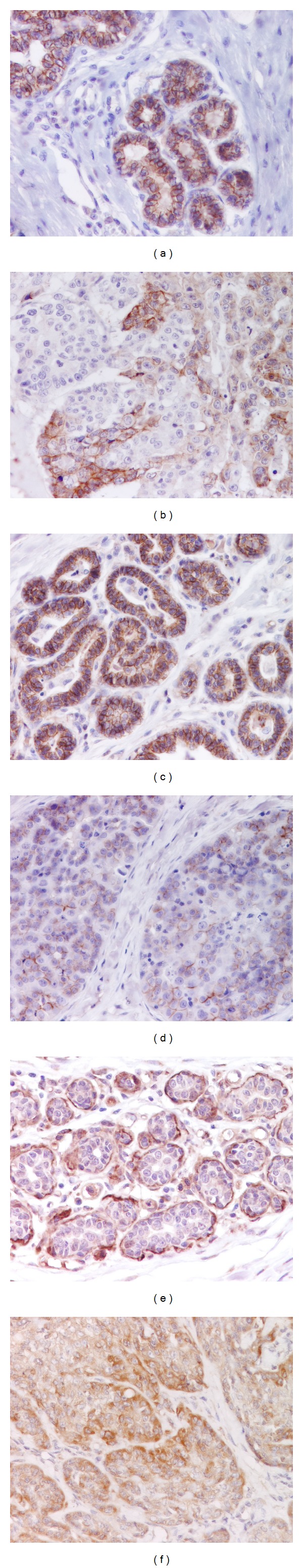
Immunohistochemical reactivity to adhesion molecules in canine mammary gland tissues. Normal mammary gland stained with antibodies to E-cadherin (a), *β*-catenin (c), and P-cadherin (e). Mammary carcinomas showing reduced expression for E-cadherin (b) and *β*-catenin (d), and aberrant expression for P-cadherin (f).

In normal canine mammary gland, E-cadherin and P-cadherin show a distinct pattern of expression. E-cadherin and *β*-catenin are expressed by epithelial cells [33–35], whereas expression of P-cadherin is restricted to myoepithelial cells (Figures 2(a), 2(c) and 2(d)), being P-cadherin a sensitive marker for this cell type [12, 36–38]. However, during lactation in humans and dogs, P-cadherin is not found at cell-cell borders, as expected for an adhesion molecule, but rather appears to be secreted by epithelial cells [37, 39].

## 4. Cadherin and Catenins in Canine Mammary**** Cancer

Despite the prolific studies in the human setting regarding cell adhesion implications in cancer, there are still few publications available in canine species. So, the specific role of cadherin-mediated cell adhesion in canine mammary cancer has not yet been fully revealed [33–35, 38, 40–45].

### 4.1. E-Cadherin

The vast majority of studies on cell adhesion involvement in tumourigenesis and invasion have focused on E-cadherin, given that this molecule is the major cadherin implicated in epithelial cell-cell adhesion, and the majority of tumours originate from epithelial cells [32]. Thus, considering that an intact adhesion complex is required for the maintenance of normal intercellular adhesion, several studies dating back to the 1990s have proposed that E-cadherin might function as a tumour and invasion suppressor molecule such that a disturbed function of E-cadherin-catenin complex in theory enhances the tumour cell invasive potential [46].

There are several lines of evidence pointing out to E-cadherin tumour/invasion suppressor function, namely, *in vitro* and *in vivo* experiments demonstrating that E-cadherin downregulation promotes tumour progression and invasion [18, 47, 48] and the presence of E-cadherin gene mutations in several human cancers, including human breast cancer [49]. In addition, the loss or reduction of the E-cadherin-catenin complex has been extensively associated with human breast cancer progression [50–56]. 

In 1997, Restucci and coworkers evaluated for the first time the immunohistochemical E-cadherin expression in canine mammary tumours, describing a reduced membranous expression in malignant neoplasia [33], especially in poorly undifferentiated cases, which was later on confirmed by other groups [35, 40], but not by others [42]. Reis et al. also found that the reduction of E-cadherin expression was significantly associated with malignancy, with all benign tumours analysed exhibiting a strong intercellular immunostaining [40].

According to our and other studies, reduced membranous expression of E-cadherin was significantly associated with tumour histological type. Solid-type carcinomas showed frequent loss of E-cadherin expression, in contrast to tubulopapillary carcinoma type [35, 40, 42].

Human studies usually document the loss of E-cadherin in lobular carcinomas, which result from a mutational inactivation of E-cadherin gene, frequently associated with the loss of heterozygosity of the other allele [57]. In such tumours, E-cadherin acts as a typical tumour suppressor gene. Recently, Ressel et al. described the morphological and immunohistochemical features of three canine mammary tumours comparable with human infiltrating lobular carcinoma, which were also negative for E-cadherin, similarly to their human counterpart [58].

A number of investigators, including our group, reported that reduced E-cadherin expression was associated with invasion (Figure 2(b)) [34, 35, 41–43], and several classic prognostic features [42], namely, proliferation [59] and lymph node metastases, suggesting this molecule as a potential prognostic marker for canine mammary cancer [35, 43]. In humans, some studies have not confirmed this relationship [36, 53, 60] or indeed have associated E-cadherin expression with lymph node metastasis [61, 62]. E-cadherin expression and function do not inevitably inhibit invasion, as it was demonstrated by Spieker and coworkers, by using an embryonic chicken heart assay [63]. Although expressing E-cadherin, canine mammary cell lines were all invasive in this *in vitro* study, which suggests that invasion depends upon other microenvironmental factors [63]. Recently, Nowak et al. found a negative correlation between E-cadherin and MMP9 expression in canine mammary carcinomas, suggesting that the loss of E-cadherin-mediated adhesion and the increase of MMP-9 could play an important role during tumour invasion [59].

The analysis of E-cadherin expression in lymph node metastases was rarely reported and several patterns of expression have been described, namely, downregulation, upregulation, or similar expression levels with regard to primary lesions [40, 64]. According to Bukholm and Nesland, there is an E-cadherin reexpression on metastatic tissues, when compared to primary tumours [65]. This is probably related to the flexibility of adhesion complexes, which might be temporarily lost in primary tumours and recovered after reaching the metastatic site [33, 65]. Probably, downregulation at primary site facilitates the detachment of cells and invasion, whereas at metastatic sites, the reexpression might allow the attachment of cancer cells [61]. This dynamic change in E-cadherin expression can be explained by promoter methylation or posttranslational regulation of cadherin-catenin complexes [32]. Recently, Pinho et al. pointed out to E-cadherin posttranslational modifications by N-glycosylation during the acquisition of the malignant phenotype, which might explain this dynamic modulation [66].

However, it is unlikely that a single molecule can determine the acquisition of a less differentiated and more invasive neoplastic phenotype and other molecules must be considered in order to understand the complex mechanisms that lead to metastasis [33].

The expression of E-cadherin complex partner, *β*-catenin, has also been evaluated in canine mammary cancer. We and others found a significant positive association between these two proteins, which is consistent with the formation of adhesion complexes on the cell membrane [35, 41, 43]. 

In addition, reduced membranous *β*-catenin expression was found to be significantly associated with high histological grade and invasion in several reports (Figure 2(d)) [35, 41], although not corroborated by Matos et al. [43]. No association has been described between reduced *β*-catenin and the presence of lymph node metastases [35, 43], which is similar to other human breast studies [53, 56, 67].

The prognostic significance of E-cadherin and *β*-catenin expression in terms of survival of dogs with mammary carcinoma is debatable. Whereas Brunetti et al. found no association with survival, our group showed that loss/reduction of E-cadherin and *β*-catenin expression is significantly associated with shorter survival times [35, 41].

Although, in general, loss of E-cadherin expression correlates with undifferentiated human breast carcinomas, the available studies differ with regard to its association with survival and its value as a prognostic marker is still controversial [10, 52, 56, 68–71]. In fact, in neoplastic cells of one of the most aggressive forms of breast cancer, such as inflammatory breast cancer, the expression of E-cadherin is consistently elevated [72]. Charafe-Jauffret et al. also confirmed E-cadherin expression in this highly metastatic carcinoma, determining that E-cadherin expression is one of the key molecules of the “inflammatory signature” [73]. The author suggested that the loss of E-cadherin expression is a transient event that allows malignant cells to invade vascular channels and tissues and once in the circulation, cancer cells reexpress E-cadherin, facilitating intercellular adhesion and enabling the formation of cohesive tumour emboli. Our group observed the same pattern of expression in canine inflammatory mammary carcinomas, which were characterized by tumour emboli strongly positive for E-cadherin (Gama et al., unpublished data).

### 4.2. P-Cadherin

Besides E-cadherin, a number of canine mammary tumours also express P-cadherin cell adhesion molecule (Figure 2(f)) [38], which corroborates previous human findings. This molecule has been detected as altered in various human cancers, with P-cadherin found to be expressed by a subset of human breast carcinomas, but its exact role in the carcinogenic process still remains unclear [16].

In canine mammary tumours, a significant association was found between P-cadherin expression and tumour histological type [38]. However, more recently, when analysing a larger tumour series, P-cadherin expression was only significantly associated with an invasive tumour phenotype, with no association with other clinicopathological variables, proliferation, or survival [35].

Our results are discordant with the majority of available studies in human breast cancer, which found P-cadherin significantly associated with several aggressive characteristics, such as high histological grade and proliferation, as well as with a poor prognosis [60, 68, 74–78], which has raised the interest on P-cadherin as a potential prognostic marker for this disease.

With regard to histological types, P-cadherin is commonly identified in canine mammary carcinosarcoma and spindle cell carcinoma subtypes, as well as in human breast medullary and metaplastic carcinomas, which evokes for a basal/myoepithelial cell histogenetic origin or line of differentiation for these tumours [38, 79, 80].

In human cancer studies, high-throughput microarray technologies allowed the distinction of breast cancer molecular subtypes (luminal A and B, HER-2 overexpressing, basal-like, and claudin-low), with basal-like phenotype significantly associated with poor prognosis [81–85]. Ever since, several immunohistochemical surrogate panels have been suggested for the identification of this subgroup [86–88], and P-cadherin is considered as one of the most sensitive biomarkers in distinguishing basal breast carcinomas [16, 78, 89]. Similar to human findings, we have recently described P-cadherin expression significantly associated with a basal-like phenotype in canine mammary carcinomas, which were characterized by poor prognostic features, such as high histological grade and proliferation, as well as with shorter disease-free and overall survival rates [90].

In our study, most luminal canine mammary carcinomas (ER positive) were P-cadherin negative [90]. P-cadherin expression was also found inversely correlated with hormonal receptor status in human breast carcinomas [75, 78, 91] and it seems to be associated with an estrogen-independent tumour growth [75]. 

A number of hypotheses have been proposed to justify the anomalous expression of P-cadherin by breast cancer cells, namely, the oncofetal properties of P-cadherin protein [37], its histogenetic origin in cap cells or acquisition of a stem cell like phenotype [68, 74]. However, although P-cadherin positive carcinomas seem to have a myoepithelial/basal-like transcriptomic programme, this reason probably does not account for every P-cadherin expressing tumour and it appears reasonable that certain molecular mechanisms would lead to the activation of P-cadherin expression during epithelial transformation [16].

In fact, a significant correlation was recently described between P-cadherin expression and hypomethylation of a specific region of the CDH3 promoter, suggesting an important regulatory role for cytosine methylation in the aberrant expression of P-cadherin in breast cancer [77]. On the other hand, the lack of ER signalling was found responsible for the increase in P-cadherin, categorizing CDH3 as an oestrogen-repressed gene and pointing to E2 as a key regulator of this cadherin [92]. In addition, it was shown that chromatin-activating modifications are also relevant in the modulation of P-cadherin gene, which suggests an additional epigenetic regulation [93].

Although the biofunctional role of P-cadherin in tumour progression is far from being fully elucidated, several *in vitro* studies using human breast [92, 94] and pancreatic [95] cancer cell lines have suggested a proinvasive role for this molecule, through its interaction with several signalling molecules, such as Rho GTPases and p120ctn [94, 95]. This proinvasive activity depends on the JMD of the cytoplasmic tail, which binds to p120-catenin [92]. Recently, it was demonstrated that P-cadherin overexpression, in human breast cancer cells with wild-type E-cadherin, induces the secretion of matrix metalloproteases, specifically MMP-1 and MMP-2 and promotes cell invasion, motility, and migration, due to a mechanism involving alterations in the actin cytoskeleton and signalling through small GTPase-binding proteins [94]. Taking into account its role in cancer cell invasion, a selective human monoclonal antibody was recently produced against P-cadherin, which is currently under Phase I clinical trials [96].

Besides breast cancer, P-cadherin has been studied in several human cancers, and it seems to behave differently depending on the cancer model [16]. In canine mammary cancer, novel investigations are welcome in order to unravel P-cadherin potential role in tumour progression. Whether it represents a useful prognostic marker or plays a causal role is still open to question.

## 5. Conclusion and Future Perspectives

Taken together, the overall findings in canine mammary cancer suggest a possible role for E-cadherin-mediated adhesion in preventing invasion and metastasis in this animal model, corroborating human breast cancer studies. Yet, results are not consensual and larger controlled studies are required in order to definitively determine cell adhesion implication in the multifaceted metastatic process, as well as the usefulness of cadherins and catenins as valuable prognostic markers and potential therapeutic targets for canine mammary carcinomas.
